# Development of strategies for effective communication of food risks and benefits across Europe: Design and conceptual framework of the FoodRisC project

**DOI:** 10.1186/1471-2458-11-308

**Published:** 2011-05-13

**Authors:** Julie Barnett, Aine McConnon, Jean Kennedy, Monique Raats, Richard Shepherd, Wim Verbeke, Jon Fletcher, Margôt Kuttschreuter, Luisa Lima, Josephine Wills, Patrick Wall

**Affiliations:** 1 Department of Information Systems and Computing, Brunel University, Kingston Lane, Uxbridge, UB8 3PH, UK; 2 School of Public Health, Physiotherapy and Population Science, University College Dublin, Woodview House, Belfield, Dublin 4, Ireland; 3 European Food Information Council (EUFIC), Rue Guimard 19, 1040 Brussels, Belgium; 4 Food, Consumer Behaviour and Health Research Centre, Department of Psychology, University of Surrey, Guildford, Surrey, GU2 7XH, UK; 5 Department of Agricultural Economics, Ghent University, Coupure links 653, B-9000 Gent, Belgium; 6 Brook Lyndhurst, Lion House, 26-28 Paddenswick Road, London, W6 0UB, UK; 7 Center for Risk & Safety Perception (CRiSP) Psychology & Communication of Health & Risk, Citadel H440, University of Twente, P.O. box 217, 7500 AE Enschede, The Netherlands; 8 Dept of Social and Organizational Psychology, Instituto Superior de Ciências do Trabalho e da Empresa, Av. das Forças Armadas, 1649-026 Lisboa, Portugal

## Abstract

**Background:**

European consumers are faced with a myriad of food related risk and benefit information and it is regularly left up to the consumer to interpret these, often conflicting, pieces of information as a coherent message. This conflict is especially apparent in times of food crises and can have major public health implications. Scientific results and risk assessments cannot always be easily communicated into simple guidelines and advice that non-scientists like the public or the media can easily understand especially when there is conflicting, uncertain or complex information about a particular food or aspects thereof. The need for improved strategies and tools for communication about food risks and benefits is therefore paramount. The FoodRisC project ("Food Risk Communication - Perceptions and communication of food risks/benefits across Europe: development of effective communication strategies") aims to address this issue. The FoodRisC project will examine consumer perceptions and investigate how people acquire and use information in food domains in order to develop targeted strategies for food communication across Europe.

**Methods/Design:**

This project consists of 6 research work packages which, using qualitative and quantitative methodologies, are focused on development of a framework for investigating food risk/benefit issues across Europe, exploration of the role of new and traditional media in food communication and testing of the framework in order to develop evidence based communication strategies and tools. The main outcome of the FoodRisC project will be a toolkit to enable coherent communication of food risk/benefit messages in Europe. The toolkit will integrate theoretical models and new measurement paradigms as well as building on social marketing approaches around consumer segmentation. Use of the toolkit and guides will assist policy makers, food authorities and other end users in developing common approaches to communicating coherent messages to consumers in Europe.

**Discussion:**

The FoodRisC project offers a unique approach to the investigation of food risk/benefit communication. The effective spread of food risk/benefit information will assist initiatives aimed at reducing the burden of food-related illness and disease, reducing the economic impact of food crises and ensuring that confidence in safe and nutritious food is fostered and maintained in Europe.

## Background

### Introduction

After a succession of food scares across Europe involving real or perceived public health effects (including Salmonella in eggs [1], BSE in beef [2] and dioxins in animal feed), consumer confidence in the safety of the EU food supply, the ability of the regulatory agencies to police the food chain, and the commitment of the food industry to produce safe food [3], was arguably at an all-time low. This precipitated a reform of EU food law [4] and led to the creation of the European Food Safety Authority (EFSA) and national food safety agencies in many Member States with consumer protection paramount. Despite the presence of these agencies, communication of food risks and benefits remains challenging, with on-going public concerns about contaminants in the food supply and technology developments (such as nanotechnology and genetically modified (GM) foods), as well as diet-related diseases (such as cardiovascular disease, obesity and diabetes) which arguably lead to greater human health impacts than food safety crises [5]. Some of these debates are conducted in the public eye, often accompanied by expressions of public and stakeholder outrage and generally characterised by intense media activity. These may in turn be linked with the collapse of segments of the food chain, damage to governments, restriction of trade and international trade disputes, as well as constraints to the development of associated food technologies [6]. Other food issues do not capture the public imagination, are associated with limited or intermittent public debate, and attract little media attention even though experts consider the evidence worthy of both attention and action. An issue core to both of these scenarios, and relevant across the EU and beyond, is the communication of risk and benefit.

The objective of this paper is to provide an overview of the background and rationale, as well as the approved study design, workplan and methodologies that will be applied within the EU-funded Seventh Framework (FP7) collaborative research project FoodRisC (2010-2013). The aim is herewith to inform the scientific and public health community about this initiative and its expected outcomes.

### Study rationale

The last thirty years have witnessed growing attention to the question of how best to communicate risk and benefit in relation to food [7] and the way in which people attend and respond to the myriad of information sources that they may encounter. Public views, understandings and concerns about food issues are related to media coverage, although this relationship is not straightforward [8]. Good communication practice seeks to bridge the divides between scientific experts, policy makers, practitioners and consumers. This is done, for example, by presenting scientific assessments to the public in understandable terms and also by ensuring that the nature of the public's concerns are known to and represented by risk/benefit managers [9]. The perception and communication of food risk thus presents an ongoing challenge to a range of stakeholders in Europe [10], particularly in light of public health concern and media attention around food crises [11]. Consumer perception and communication relating to benefits is equally challenging, for example, to ensure adequate levels of consumer protection around dietary recommendations and nutrition and health benefit claims. In addition there are particular challenges where the simultaneous communication of risk and benefit is required [12].

### The traditional model of risk communication

**Figure 1:**
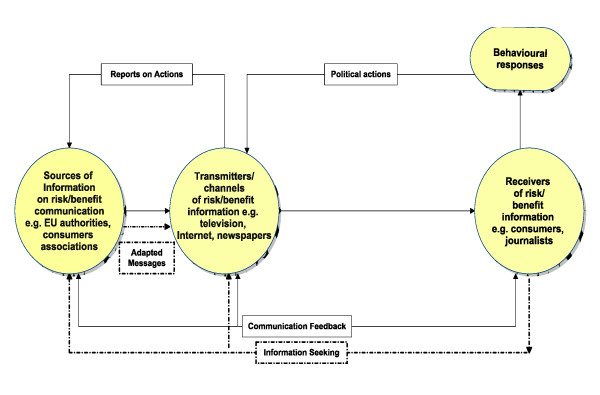
**Communication of Food Risk/Benefit: a Source-Transmitter-Receiver framework**. This figure is adapted from Renn, O (2008) Basic Concepts and Challenges of Risk Communication, In O. Renn, Risk Governance: Coping with Uncertainty in a Complex World, London: Earthscan.

The dominant understandings of risk communication continue to be aligned around the traditional model of information transfer between sources, transmitters/channels and receivers [13] and, though criticised as being overly mechanistic, it continues to provide a useful springboard for depicting and analysing the risk communication process [14]. In Figure 1 the traditional model has been adapted to draw attention to the potentially active role of recipients of information, and the way in which their feedback and information seeking can be received by the information source and, in the light of this, messages subsequently adapted. It is vital to understand the contexts in which there is potential for active consumer involvement in interpreting, affecting and even creating risk/benefit communications.

### Risk and benefit communication in relation to food

In communicating food risks it may be vital in many instances to take account of the overall configuration of both risk and benefit [15]. Risk communication around food is arguably a unique area in that the benefits it provides are necessary for survival [16]. There are of course a range of possible relationships that may exist between food risk and food benefit. Different positive and negative effects exist with all food; one example which has already received some research interest is that of oily fish, with its associated health risks (mercury) and benefits (Omega 3) [17,18]. These different configurations of risk and benefit - and the degree of uncertainty attached to these - have implications for the required actions of risk communicators, for example in terms of the required speed of response or the degree of required consumer involvement. In order to be able to develop common approaches for communicating coherent messages across Member States, it is vital to appreciate the communication implications of food risk/benefit configurations of risk for the more routine hazards as well as for food crises. Still finer distinctions between risk/benefit configurations will have implications for communication. For example the EU funded BRAFO project [19] compares food containing sources of risk that have no direct health benefits (e.g. pesticides) and those that do (e.g. micronutrients) using a common scale of measurement. Finally, large-scale food technologies such as nanotechnologies and genomics are promoted in terms of the opportunities and potential benefits they afford. The development trajectory of GM foods illustrates the need for those responsible for communication of the benefits to understand the barriers to risk communication and of the potential value of communicating through trusted sources and multiple channels.

### The role of social media in food risk/benefit communication

Although enormous progress has been made in understanding the determinants of risk perception and in identifying the necessary ingredients of effective food risk and benefit communication, this has not been matched with the development of efficient and appropriate tools. Very little work has been done examining the implications of the explosion of new media and web technologies for food risk/benefit communication. This growth in new social media offers particular potential for improving the communication of food risk and benefit but must be considered alongside the classical media channels, the more traditional role of journalists as well as those whose access to new media is limited. Increasing numbers of people however are using new media for information access and onward distribution of news. Online communities, social network sites, blogging, micro-blogging (e.g. Twitter) and e-mail groups provide a 24 hour, 365 days per year 'alert' service for their members who in turn update others in their own networks. It is also vital to consider the way in which new social media interact with the more established media and the traditional role of journalists. Many traditional news services increasingly provide information via online web sites and blogs and engage in the concept of content generated by 'citizen journalists'. 'Citizen Journalism' is known as public or participatory journalism and is the act of non-professionals playing an active role in the process of collecting, reporting, analyzing and disseminating news and information [20].

Even when the communication context requires authorities to issue 'top down' messages, consumers are not simply passive receivers of information. 'Active sense making' often accompanies the reception of information. Consumers will vary in their capacities to decode information and in how responsive they are to the information that they are given. Greater understanding is needed of three information-relevant dimensions of consumer behaviour - responding, seeking and deliberating - and of the impact upon each of them of multiple, contested or uncertain communications. The social marketing literature brings further insights here in considering how best to target particular subgroups of the population with particular messages. In relation to food, in addition to differences between different countries [21], gender is a crucial socio-demographic parameter of segmentation [22], as men and women have different social practices around consumption, household behaviour and responsibility around food issues [23]. Furthermore, there are differences between men and women; in their vulnerability to food risks (e.g. hormonal changes during pregnancy make women more susceptible to Listeria infections) and micronutrient deficiencies (e.g. iron); in patterns of risk perception [24] and their patterns of dietary behaviour [25] and experiences of communication technologies [26].

## Methods/Design

The FoodRisC project http://www.foodrisc.org is a three year project which started in June 2010 and aims to progress beyond the current state of the art in a number of these areas. Notably it will provide research evidence on the role of information seeking and questioning on the part of consumers and critical trust in food risk communication across Europe, through information seeking and deliberation. By integrating novel measurement methods that bring together qualitative and quantitative self report data with behavioural measures of attention to risk and benefit information, this project will extend the state-of-the-art in measurement of risk and benefit perception. This research will lead to the development of a toolkit that will (i) integrate theoretical models and new measurement paradigms, (ii) develop a systematic characterisation of both the implications of different risk/benefit configurations and of the potential of new media, (iii) build on social marketing insights around consumer segmentation. Through dissemination and training, this toolkit will directly improve current practice in food communication among national and international policy groups.

The specific objectives of the FoodRisC project are to:

1. Characterise key configurations of food risk/benefit relationships and the consequent implications for communicators.

2. Make recommendations about the unique potential of new social media and provide practical guidance as to how risk communicators can best use these media for the communication of food risk and benefit.

3. Characterise consumers and the ways in which consumers *respond *to information about food risk and benefit, taking into account gender and other relevant socio-demographics as well as important qualities of the information itself and the context in which it is given, e.g. the expression of uncertainty, existence of multiple information sources, contestation or conflict.

4. Investigate and characterise the role of information *seeking *in relation to food risk and benefit, taking into account different stimuli and contexts.

5. Characterise the potential use and role of *deliberative engagement *in food risk and benefit communication.

6. Propose a strategy alongside the necessary tools for effective communication of coherent messages across the Member States, which could also support the implementation of EU policy initiatives.

In order to identify barriers to effective communication and to develop common approaches for communicating coherent messages across Europe this project will provide new evidence in relation to five broad areas:

• Characterisation of food risk and benefit issues and the consequent communication implications

• Potential role of new social media in communicating food risk/benefit

• How consumers respond to information they perceive as uncertain, contested or confusing and to develop relevant segmentation criteria

• Applicability of the concept of information seeking to the design of food risk/benefit communications

• Developing practical ways in which consumer sense making and deliberation can be taken into account in order to provide substantive benefits to stakeholders in developing communications

These areas of research will be addressed in the following order:

### 1. Development of the FoodRisC framework

A recent review of the state of the art in food risk communication [16] has suggested the value of tailoring communication strategies to different profiles of food risk and benefit. One core strand of the current project is to take this recommendation forward by systematically characterising the communication requirements of different food risk/benefit configurations. Food risk perception and communication studies have primarily been located around 'food scares' with limited consideration of communication around the more everyday examples of food risks and benefits [27].

### 2. Use of new media

The role of new 'social' media has thus far been peripheral to consideration of the communication of risk. Rather the focus has been upon traditional instruments of risk communication such as brochures and leaflets, information videos, and exhibitions [7]. However the reach of innovative communication technologies is increasing exponentially and the need to embrace the potential they provide has recently been identified as one of the key challenges of the coming years [28]. This project will provide a systematic examination of the potential for their use across Europe and practical guidance as to how they can best be used both to understand what concerns people have and how they are making sense of communications as well as how best to use social media as a communication tool.

### 3. Testing of the framework

Certainly a key challenge for those charged with communication of risk/benefit is to systematically take into account the perspectives of those with whom they wish to communicate. A basic dilemma in meeting this challenge is to be informed by the perspectives of those at whom the communication is aimed whilst at the same time fulfilling the responsibility to issue relevant, speedy, authoritative communications where necessary. One innovative way in which FoodRisC addresses this challenge is through the development at European level of an online deliberation tool that has already undergone extensive piloting in the UK [29]. The key properties of this tool are to enable people to engage with information on their terms, to facilitate the expression of questions and comments, and to address these. In a move away from 'event-based' deliberation, this tool engages people in a way that approximates the more everyday processes of information seeking and sense making. This tool, known as EnGauge, not only allows for the analysis of the textual material participants provide but links this with precise behavioural indicators of online behaviour, for example, what material has been accessed and for how long.

**Figure 2:**
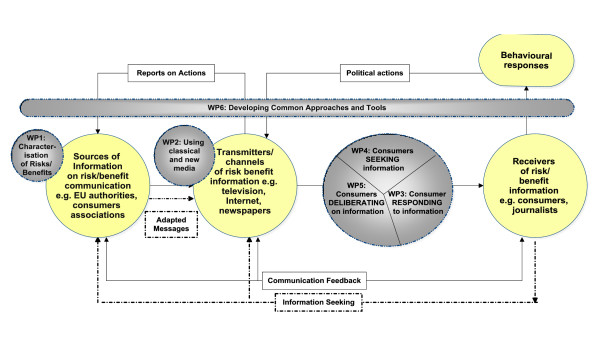
**Relationship between the initial communication framework, the FoodRisC research objectives and their associated work packages**.

Figure 2 depicts the relationship between the initial communication framework and the research objectives and their associated work packages.

#### Work plan and methodologies

**Figure 3:**
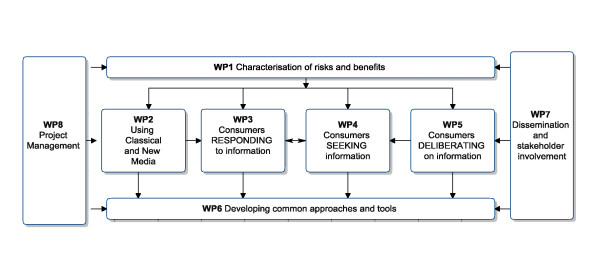
**The overall structure of the FoodRisC work plan**.

Developing a common approach to the communication of food risk and benefits is complex and requires a multi-method approach drawing on relevant theory and methods from a range of disciplines. The overall work plan is depicted in Figure 3 and the project consists of eight work packages.

##### Characterisation of risks and benefits

Characterisation of food risk/benefit configurations will involve exploration and mapping of the variety and diversity of target population groups, relevant stakeholders, communication tools, information sources, media channels and risks/benefits currently and potentially involved in risk/benefit communication in the food chain. Identifying the parameters of current food risk/benefit communication models in Europe provides baseline information on: a) the variety of stakeholders and population sub-groups involved in food risk/benefit communication, b) the perceived parameters of food risk/benefits, which may include nanotechnology, gene-technology, cloning, emerging pathogens, functional foods etc. among the various stakeholders, c) the current communication tools/sources/channels/approaches used for food risk/benefit communication nationally and across Europe. This work will investigate consumers' knowledge and use of risk/benefit communications and preferred communication routes and tools as well as perceived barriers to effective risk and benefit communication. In addition the opinions of food chain stakeholders and experts regarding food risk/benefit communication tools and information routes as well as perceived barriers to effective risk and benefit communication will be explored. This work will be conducted in work package one (WP1) and the results of this work package (WP) will inform the systematic identification of a range of relevant risk/benefit issues that will be explored in WPs 3-5 and will inform the development of these WPs.

##### Using classical and new media in food communication

The FoodRisC project will investigate media involvement in communication in the food chain and will identify barriers to efficient food communications and potential use of new and classical media channels to communicate food risks/benefits efficiently. This work is concerned with the synergy and inter-relationship between classical media channels and new information routes/technologies in providing effective and efficient information sources for food risk/benefit communication.

The role and use of classical and new media in food risk/benefit communication by both traditional and citizen media journalists will be investigated post-event as well as in the context of a real-time food alert. Different communications will be tracked and monitored, comparing new media and classical media handling via tracking software, analysis of postings and direct surveys of contributors to online information communities. This will enable evaluation of who influences whom, how they are influenced, and what sources of information were used in food crisis/non-crisis situations. This work will provide data on the information and support that is available to journalists to enable them to report food risk/benefit issues accurately. Data on what sources, data validation, use and accreditation of information sources are required and/or available to online and traditional journalists will also be generated. This work will take place within WP2 and the results of this WP will also inform the work of WP3-5.

##### Investigating the role of consumers in food risk/benefit communication

Key consumer alignments to the communication of risk and benefit information will also be explored in the FoodRisC project. This research will comprise three work packages (see Figure 2) and will involve a) characterisation of *how consumers respond to information (Work package 3) *b) characterisation of *consumers' information seeking behaviour (Work package 4) *and c) exploration of the role and potential use of *deliberative engagement in food risk/benefit communication (Work package 5)*.

A Pan-European web-based survey will collect data that will be used to map consumers' food risk/benefit perceptions in WP3 and the modelling of information seeking behaviour in WP4. Characterising consumers and their responses to communication of information about food risks/benefits will enable segmentation of European consumers in relation to different risk/benefit scenarios, across different media, and in crisis and non-crisis situations. This will inform an understanding of how specific messages to population subgroups should be tailored. This work will determine consumer responses to food risk/benefit scenarios and predictors of risk/benefit perceptions in European consumers.

Research on the role of information seeking in food risk/benefit communication will explore the stimuli and contexts which induce individuals to look for information relating to food risk/benefit. This work will also investigate the implications of consumers using a broad range of information sources. This work will provide a) quantitative insight into the main determinants of risk and benefit information seeking, b) consumer segmentation in relation to preferences for use of communication channels, c) experimental evidence on the ways in which online information seeking strategies are affected by the provision of risk and benefit information, and d) details on what information consumers seek from official bodies about food risk/benefits in crisis and non-crisis situations.

The final set of empirical studies will take place within WP5 and will investigate the role of deliberation in developing risk and benefit communication strategies. This work will consider how consumers make sense of information in the context of two-way information exchange and deliberation, and will involve the development of a web-based communication tool. The overall objective of this work is to develop and test a tool that aims to facilitate the efficient and effective deliberative engagement between communicators and particular groups of consumers whose views and concerns they wish to engage with. It will provide communicators with access to consumer reasoning around risk and benefit and provide concrete measures of the extent to which consumers attend to and reflect upon the information with which they are provided.

##### Development of common approaches and tools for optimal food risk/benefit communication

The previous empirical research (WP 1 - 5) will then be brought together in the development of common approaches and tools for optimal food risk/benefit communication across Europe. This work will happen in WP6 and will involve the development of a set of tools designed to enable coherent communication of food risk/benefit information in Europe. A FoodRisC media channel choices framework will be developed to assist best practice in food risk/benefit communication through classical and new media routes. In particular the potential for using various new media routes will be outlined along with their strengths and potential weaknesses for use with various population groups and in different communication contexts. Other FoodRisC outputs will include an exploration of the potential and best practice use of the deliberative engagement tool, the development of a FoodRisC process design tool to enable informed choices about effective targeted communication of food risk/benefit across the Member States as well as the development of the FoodRisC method selection tool to enable informed choices about methods to elicit consumer perspectives on risk benefit issues. These tools will be evaluated by relevant stakeholders in a project workshop before they are finalised. A final workshop will disseminate the final toolkit and results to the project's target audiences. To extend the reach of this event, it will also be available as a webinar and made freely available to download. Following this workshop the final report will detail project outcomes and their applicability for policy officials, practitioners and communicators across the private and public sectors.

##### Project Management and Dissemination

In addition to the research work packages there are two additional work packages dealing with the project management and dissemination and exploitation of the project results. Stakeholder involvement, dissemination and exploitation of results will be on-going throughout the life of the FoodRisC project (*Workpackage 7*). The comprehensive insights gathered in the research-based WPs will be disseminated to stakeholders, and their feedback sought and used to inform the development of an evaluated toolkit to enable communication of coherent messages across Europe. This work will proactively promote the use of results from the project among the target groups: opinion leaders/regulators, media, food and drinks industries, RTD performers, consumer associations and wider society. Dissemination of project results will be to a wider audience, of relevant stakeholders at European level, including policy makers, opinion leaders, food and drink industry and SMEs, communication agencies and other communicators, scientists, professional associations, consumer organisations and NGOs, media, and the broader public.

Finally management of the project will comprise the coordination and management of all aspects of the project, in accordance with the EC grant agreement and ensure maintenance of the consortium agreement (*Workpackage 8*).

##### Ethical issues

The highest ethical standards applicable to social sciences research will be adhered to throughout all research activities planned in the FoodRisC project. This study protocol has undergone two levels of ethics review. Firstly the study proposal was screened and approved by the Ethics Review Panel of the European Commission as part of the project evaluation phase in 2009. In addition the study has been granted full ethics approval by the Human Research Ethics Committee of University College Dublin, Ireland (ethics approval number LS-11-04). Ethical issues pertaining to the planned research with healthy human volunteers include: informed consent procedures, protection of privacy, and data protection. All information will be stored in anonymous and non-identifiable formats and each of the studies will commence only after obtaining ethical approval from competent ethics committees. In those cases where data collection will be subcontracted to professional market research agencies, it will be ensured that these abide the ESOMAR code of conduct for social sciences research.

## Discussion

Through a series of interlinked WPs the FoodRisC project will explore and investigate consumer perceptions, preferences and current practices in the area of food risk/benefit communication. This project brings together social scientists, nutritionists, communication and social media experts to work together to investigate the issue of communicating food risks/benefits across Europe. Additionally, the 14 partners from nine countries will remain attuned to the emerging food issues and challenges through their partnership with a Stakeholder Advisory Board containing the European Food Safety Authority (EFSA), The Confederation of the Food and Drink Industries of the European Union (CIAA), EU Food Policy and others. The array of project partners in the FoodRisC consortium provides representation from a geographical spread across Europe as well as diversity in degrees of social media usage/exposure, in experience of and exposure to food risk crises, and in governance structures for food related communication and public health policies. The pan-European nature of the FoodRisC research programme will provide a comprehensive dataset on the issues relating to food risk/benefit communication in Europe. Involvement of consumer and SME organisations will enhance the scope and reach of this research.

The FoodRisC project aims to improve current practice in food communication across Europe through the development of a toolkit which will enable coherent communication. Development of the FoodRisC toolkit will integrate theoretical models and new measurement paradigms as well as building on social marketing insights around consumer segmentation. By integrating novel measurement methods bringing together qualitative and quantitative self-report data with, uniquely, behavioural measures of attention to risk information, this project will extend the state of the art in measurement of risk perception. Differing from the traditional approach to studying risk perception, this project investigates not only how consumers respond to information provided to them, but it also explores information seeking behaviour and the role of deliberation in food risk/benefit communication which is a unique combination in the food risk domain.

An explosion of the use and availability of new social media has led to a dramatic change in traditional communication. The FoodRisC project aims to explore the role of new social media, as well as traditional media, in communication of food issues and provide best practice guidelines for new social media in food risk/benefit communication. This work will explore both consumers' and stakeholders' views and use of different media channels as well as testing the role and scope of new media channels in food communication. Thus far, there has been little use of social media in the exchanges between public health policy makers, other stakeholders, and the public around food risks and benefits. This project will map out this domain which is likely to become of increasing importance over the next few years and provide guidance as to how social media can be used to provide intelligence around emerging public debates, to provide influential advice both in short term crisis situations and in the longer term.

The FoodRisC project offers a unique approach to the investigation of food risk/benefit communication and, in reaching its potential, will provide a coherent approach and practical guidance for stakeholders in developing responsive and meaningful communication of food risks and benefits across Europe.

## Competing interests

The authors declare that they have no competing interests.

## Authors' contributions

JB, AMC, RS, MR and JK designed the overall study structure. JB and AMC drafted the manuscript. All co-authors designed different work packages of the FoodRisC project and provided comments on the draft manuscript. All authors read and approved the final manuscript.

## Pre-publication history

The pre-publication history for this paper can be accessed here:

http://www.biomedcentral.com/1471-2458/11/308/prepub

## References

[B1] Miller D Reilly J Making an issue of food safety: the media, pressure groups, and the public sphere Eating agendas: food and nutrition as social problems 1995 305 336

[B2] Phillips L Bridgeman J Ferguson-Smith M The BSE Inquiry: the report UK Government 2000

[B3] Vos E EU food safety regulation in the aftermath of the BSE crisis Journal of Consumer Policy 2000 23 3 227 255 10.1023/A:1007123502914

[B4] Commission of the European Communities: White Paper on Food Safety 2000

[B5] Foster R Lunn J 40th Anniversary Briefing Paper: Food availability and our changing diet Nutrition Bulletin 2007 32 3 187 249 10.1111/j.1467-3010.2007.00648.x

[B6] Kasperson J Kasperson R Pidgeon N Slovic P Pidgeon N, Kasperson R, Slovic P The social amplification of risk: assessing fifteen years of research and theory The social amplification of risk 2003 Cambridge Univ Pr

[B7] Renn O Corporation E Risk governance: coping with uncertainty in a complex world 2008 Earthscan London

[B8] Vilella-Vila M Costa-Font J Press media reporting effects on risk perceptions and attitudes towards genetically modified (GM) food Journal of Socio-Economics 2008 37 5 2095 2106 10.1016/j.socec.2008.04.006

[B9] Powell D Leiss W Mad cows and mother's milk: the perils of poor risk communication 1997 McGill Queens Univ Pr

[B10] Directorate-General for Health & Consumers: Future Challenges for EU Health and Consumer Policies European Commission 2008

[B11] Knowles T Moody R McEachern M European food scares and their impact on EU food policy British Food Journal 2007 109 1 43 67 10.1108/00070700710718507

[B12] Cope S Frewer LJ Houghton J Rowe G Fischer ARH de Jonge J Consumer perceptions of best practice in food risk communication and management: Implications for risk analysis policy Food Policy 2010 35 4 349 357 10.1016/j.foodpol.2010.04.002

[B13] Shannon C Weaver W The mathematical theory of communication 1949 Urbana, IL. University of Illinois Press

[B14] Renn O Basic Concepts and Challenges of Risk Communication Risk governance: coping with uncertainty in a complex world 2008 London: Earthscan

[B15] Verbeke W Vanhonacker F Frewer L Sioen I De Henauw S Van Camp J Communicating risks and benefits from fish consumption: Impact on Belgian consumers' perception and intention to eat fish Risk Analysis 2008 28 4 951 967 18627545 10.1111/j.1539-6924.2008.01075.x

[B16] Lofstedt R How can we Make Food Risk Communication Better: Where are we and Where are we Going? Journal of Risk research 2006 9 8 869 890 10.1080/13669870601065585

[B17] Sioen I Van Camp J Verdonck F Verbeke W Vanhonacker F Willems J De Henauw S Probabilistic intake assessment of multiple compounds as a tool to quantify the nutritional-toxicological conflict related to seafood consumption Chemosphere 2008 71 6 1056 1066 10.1016/j.chemosphere.2007.11.025 18155748

[B18] Sioen I Bilau M Verdonck F Verbeke W Willems J De Henauw S Van Camp J Probabilistic intake assessment of polybrominated diphenyl ethers and omega 3 fatty acids through fish consumption Molecular Nutrition & Food Research 2008 52 2 250 257 10.1002/mnfr.200700109 18186100

[B19] Hoekstra J Hart A Boobis A Claupein E Cockburn A Hunt A Knudsen I Richardson D Schilter B Schütte K BRAFO tiered approach for benefit-risk assessment of foods Food and Chemical Toxicology10.1016/j.fct.2010.05.049 20546818

[B20] Bowman SWC Lasica JD We Media: How audiences are shaping the future of news and information 2003 The Media Center at the American Press Institute

[B21] Hohl K Gaskell G European public perceptions of food risk: cross-national and methodological comparisons Risk Analysis 2008 28 2 311 324 10.1111/j.1539-6924.2008.01021.x 18419651

[B22] Kjærnes U Trust and distrust: cognitive decisions or social relations? Journal of Risk research 2006 9 8 911 932 10.1080/13669870601065577

[B23] Jackson P Changing families, changing food 2009 Palgrave Macmillan

[B24] Dosman D Adamowicz W Hrudey S Socioeconomic Determinants of Health and Food Safety Related Risk Perceptions Risk Analysis 2001 21 2 307 318 10.1111/0272-4332.212113 11414539

[B25] Wardle J Haase A Steptoe A Nillapun M Jonwutiwes K Bellisie F Gender differences in food choice: the contribution of health beliefs and dieting Annals of Behavioral Medicine 2004 27 2 107 116 10.1207/s15324796abm2702_5 15053018

[B26] Colley A Gale M Harris T Effects of gender role identity and experience on computer attitude components Journal of Educational Computing Research 1994 10 2 129 137 10.2190/8NA7-DAEY-GM8P-EUN5

[B27] Fischer A De Vries P Everyday behaviour and everyday risk: An approach to study people's responses to frequently encountered food related health risks Health, Risk & Society 2008 10 4 385 397 10.1080/1369857080216644921568017

[B28] Marler B Marler's Ten Top Food Safety Challenges for 2009 2009

[B29] Barnett J On Line Deliberative Engagement: A Pilot Study Research Report 2008 London: The Wellcome Trust

